# Diagnosis and Clinical Implication of Left Ventricular Aneurysm in Hypertrophic Cardiomyopathy

**DOI:** 10.3390/diagnostics13111848

**Published:** 2023-05-25

**Authors:** Errico Federico Perillo, Grazia Canciello, Felice Borrelli, Gaetano Todde, Massimo Imbriaco, Leopoldo Ordine, Salvatore Di Napoli, Raffaella Lombardi, Giovanni Esposito, Maria-Angela Losi

**Affiliations:** Department of Advanced Biomedical Sciences, Federico II University, 80131 Naples, Italyfeliceborrelli@yahoo.it (F.B.); salvatore.dinapoli@unina.it (S.D.N.);

**Keywords:** hypertrophic cardiomyopathy, left ventricular aneurysm, anticoagulation, arrhythmias

## Abstract

Hypertrophic cardiomyopathy (HCM) is a genetic disease with heterogeneous clinical presentation and prognosis. Within the broad phenotypic expression of HCM, there is a subgroup of patients with a left ventricular (LV) apical aneurysm, which has an estimated prevalence between 2% and 5%. LV apical aneurysm is characterized by an area of apical dyskinesis or akinesis, often associated with regional scarring. To date, the most accepted pathomechanism of this complication is, in absence of coronary artery disease, the high systolic intra-aneurysmal pressure, which, combined with impaired diastolic perfusion from lower stroke volume, results in supply–demand ischemia and myocardial injury. Apical aneurysm is increasingly recognized as a poor prognostic marker; however, the efficacy of prophylactic anticoagulation and/or intracardiac cardioverted defibrillator (ICD) in improving morbidity and mortality is not yet clearly demonstrated. This review aims to elucidate the mechanism, diagnosis and clinical implication of LV aneurysm in patients with HCM.

## 1. Introduction

**Figure 1:**
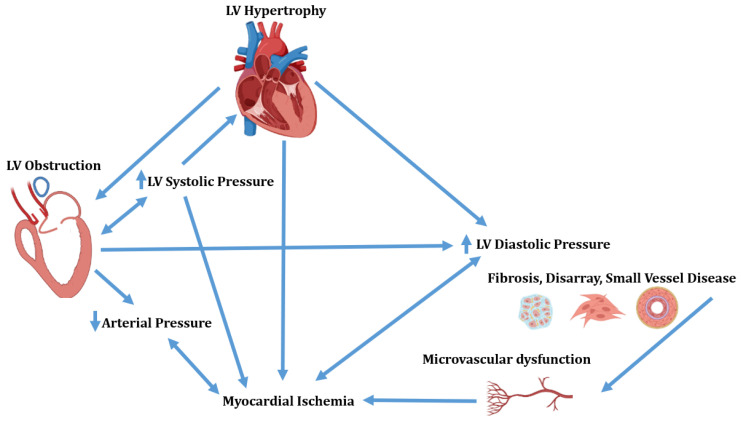
Complex interplay of morphological and functional abnormalities in hypertrophic cardiomyopathy. LV = Left ventricular.

Hypertrophic cardiomyopathy (HCM) is a myocardial disease defined in the presence of increased thickness of left ventricular (LV) wall, which is unexplained by abnormal loading conditions. The disease presents a complex spectrum of morphological and functional abnormalities. In fact, the interplay between LV hypertrophy, obstruction, interstitial fibrosis, disarray and small vessel disease results in LV obstruction, diastolic dysfunction and myocardial ischemia (Figure 1) [1].

Although many mechanisms are responsible for myocardial ischemia, the exact mechanism linked to wall thinning and the eventual aneurysm formation is not well understood. LV aneurysm is present in less than 5% of HCM patients [2,3]. Patients with LV aneurysm show more symptoms, ventricular arrhythmias and thromboembolic events than patients without it [4,5,6,7]. However, the increased risk of embolic events and of sudden cardiac death (SCD) is not universally accepted [8,9,10,11,12]. This review aims to elucidate LV aneurysm development, diagnosis and clinical implication in HCM patients.

## 2. Mechanism of LV Aneurysm Formation in HCM

**Figure 2:**
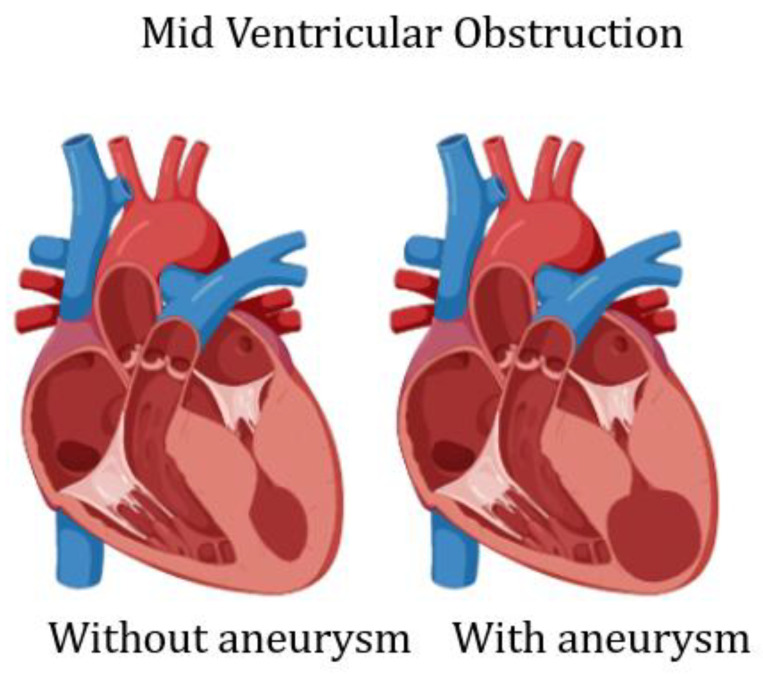
Scheme of left ventricular mid cavity obliteration, without and with aneurysm.

The term aneurysm is generally applied to the bulging or outpouching of an area of weakened myocardial wall. In coronary artery disease, a transmural myocardial infarction (MI), inducing full wall replacement by fibrous tissue, leads to the formation of a LV aneurysm. This inert portion cannot take part in the contraction and herniates outward during systole. In HCM, the presence of a LV aneurysm, in absence of coronary artery disease, has been described in less than 5% of patients [4,5,6,7]. More than 30 years ago, a sustained monomorphic ventricular tachycardia (VT) and apical LV aneurysm was reported in two individuals [1]. In one of these patients, VT arose from the lateral border of the aneurysm; after this description, apical aneurysm was described in few case reports. However, with the widespread use of cardiac magnetic resonance (CMR), there has been a renewed interest in its mechanism of formation and prognostic relevance [1]. Now, we know that, similarly to transmural MI, in HCM, the LV aneurysm wall is characterized by transmural fibrosis [13,14,15]. Myocardial scarring involving LV septum, LV free wall and one or both papillary muscles has been commonly described in necropsy studies [13,14] and in some HCM anecdotical cases [15]. Thus, replacement fibrosis, even in the absence of significant coronary atherosclerosis, is a frequent finding in HCM and is the main histopathologic finding of LV aneurysmatic wall [4,14]. Only in one case report, the cause of LV aneurysm has been ascribed to a circumferential mid ventricular contraction ring [16]. The genetic background predisposing to LV aneurysm formation has been suggested in an interesting paper by Zenovich et al., reporting identical twins with HCM and LV aneurysm, although the two twins were not genotyped [17]. Additional publications suggest that a specific mutation, influencing the LV hypertrophic pattern, could result in the development of transmural fibrosis [18,19,20,21]; however, there is no evidence that specific mutations are linked to development of mid ventricular obstruction (MVO), which seems particularly important in the development of LV aneurysm [8,22,23,24,25,26] (Figure 2).

**Figure 3:**
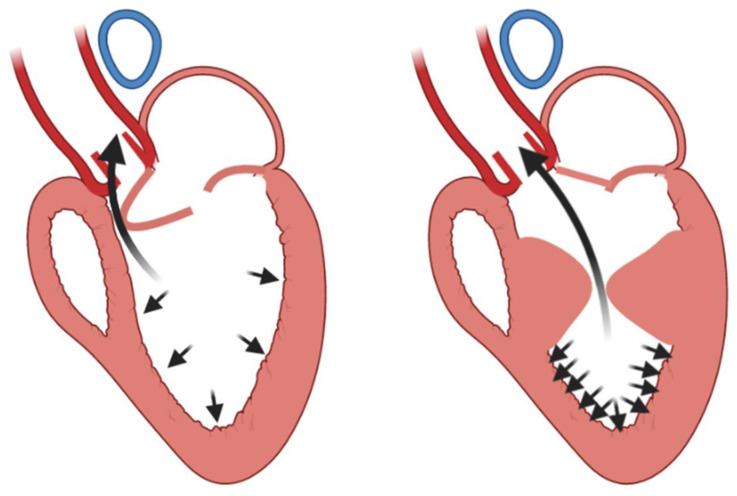
Left ventricular apex stress/strain differences between subaortic obstruction (**left** panel) and mid ventricular obstruction (**right** panel) [27].

The mechanism of LV aneurysm formation in MVO is well demonstrated in a simulation study [27]. The authors proposed three idealized finite element models to compare the stress/strain on LV apex of healthy subjects and of patients with different anatomical myocardial hypertrophy (subaortic and mid ventricular obstruction). The idealized model helped identify the position of hypertrophy as an independent factor of the genesis of apical aneurysm: the stress/strain on LV apex in the mid ventricular obstruction model was significantly higher than that in the subaortic obstruction model (Figure 3). 

The obtained results suggested that the MVO significantly increased myofiber stress in the LV apex, which might directly initiate apical aneurysm formation.

The role of MVO in the genesis of LV aneurysm was confirmed recently by Sherrid et al. [8] in a clinical study. In a large series, the authors demonstrated that HCM patients with aneurysm have a higher prevalence of MVO obstruction than those without. In addition, the study showed that patients with LV aneurysm had smaller areas measured in short axis at the mid ventricular level and larger papillary muscle heads, further suggesting that the presence of increased gradients in this LV area facilitates aneurysm development.

The increased stress at the apical level in the presence of MVO, together with small vessel disease, contributes to myocardial injury of the LV apex, as defined by the fourth universal definition of MI, i.e., increased c-troponin values > 99th percentile URL [28]. However, although highly sensitive cardiac troponin T is elevated in a significant number of HCM patients and has been proposed as a valuable marker of myocardial injury in HCM [29], there are no data linking cardiac troponin T levels and LV aneurysm formation.

As we reported above, increased interstitial fibrosis is one of the morphological characteristics of HCM; however, replacement fibrosis is not an infrequent finding in HCM. Interstitial fibrosis probably results from sarcomeric dysfunction due to mutation, which acts on local profibrotic factors, whereas replacement fibrosis seems to have an ischemic origin.

Basing on these data, to date, the most accepted hypothesis on LV aneurysm development is the following: the high systolic intra-aneurysmal pressures combined with impaired diastolic perfusion from lower stroke volume result in supply–demand ischemia and myocardial injury. These unique circumstances imposed by MVO explain the susceptible location of the apex for aneurysm formation and explain why aneurysms are not seen in other LV regions [1,8].

Other mechanisms proposed as causes of myocardial scar formation in HCM include coronary artery spasm, myocardial bridging and oxygen supply/demand mismatch [30,31]. Although the incidence of myocardial bridging is higher in patients with HCM than in the general population, its role in angina or myocardial injury, or both, nevertheless remains unclear [30].

**Figure 4:**
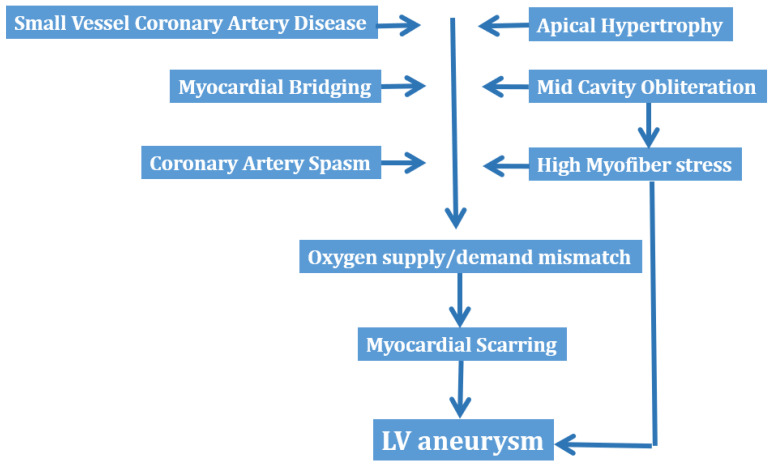
Mechanisms of left ventricular aneurysm formation in hypertrophic cardiomyopathy. LV = left ventricular.

Figure 4 summarizes the mechanisms, which have been advocated in the development of LV aneurysm in HCM.

## 3. Diagnosis of LV Aneurysm in HCM

**Figure 5:**
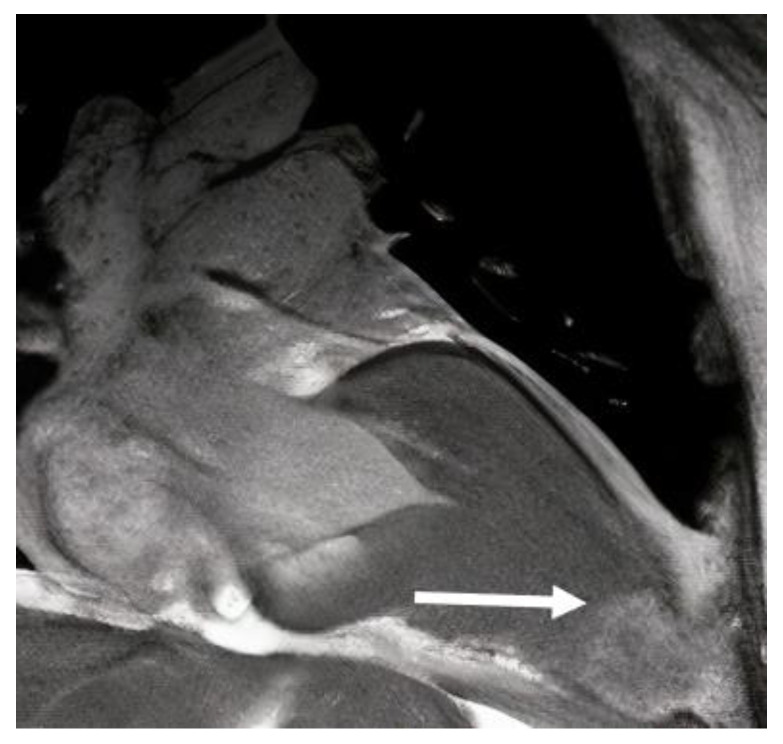
Cardiac magnetic resonance long-axis view, showing left ventricular aneurysm (arrow) in a patient with HCM.

**Figure 6:**
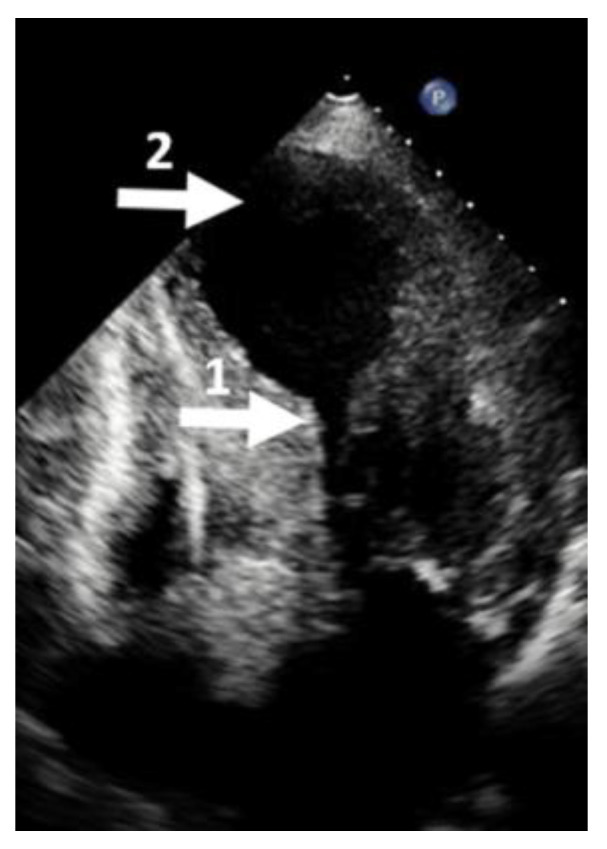
Echocardiographic four-chamber view. (1) Mid cavity obliteration; (2) left ventricular aneurysm. This picture is called the hour-glass appearance.

The reported prevalence of LV aneurysm in the HCM population is low (2–5%) [7], although this may be an underestimate, since 2D echocardiography is often unreliable in detecting smaller aneurysms compared to the greater diagnostic sensitivity of CMR (Figure 5 and Figure 6). 

To date, CMR should be executed in all patients with diagnosed or suspected HCM, in that it provides three-dimensional tomographic cardiac imaging with high spatial and temporal resolution in any plane and without ionizing radiation. CMR has unique strengths, which make it particularly well suited for providing detailed characterization of the HCM phenotype, and therefore, it can aid in the diagnosis and potentially offer prognostic information. In addition, CMR is the gold standard technique for quantification of ventricular volumes, mass and function. A further advantage of CMR is its ability to characterize myocardial tissue. The implementation of CMR in the management of HCM patients resulted in an increased number of LV aneurysms, directing attention to this unfavorable LV rearrangement.

**Figure 7:**
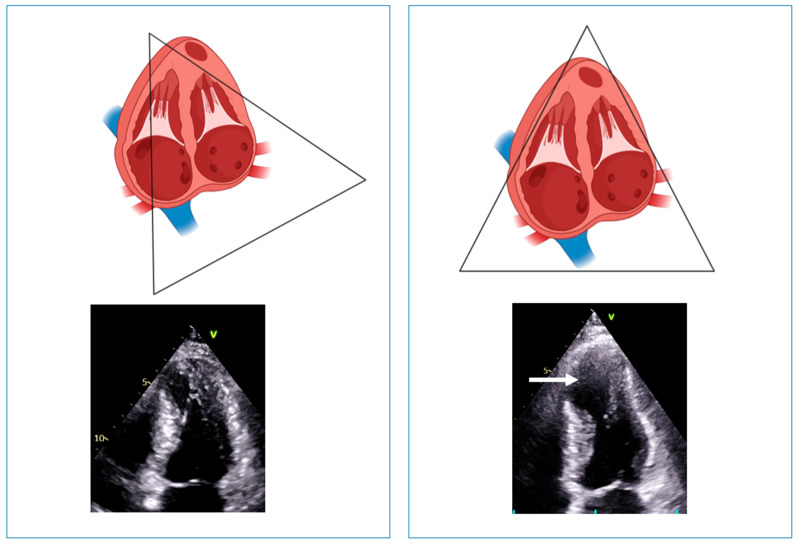
**Left** panel: example of foreshortening of the left ventricular apex. The systolic frame shows a well-contracting apex, without evidence of aneurysm. **Right** panel: positioning the probe one intercostal space lower (i.e., more toward the apex) and laterally; the left ventricular length increases, and an aneurysmatic apex appears (arrow).

In a large study including more than 1000 HCM patients, echocardiography identified LV aneurysm in only 16 of the 28 patients with definitive diagnosis by CMR. In these 16 patients, the aneurysms identified by both imaging techniques were either medium or large; among the remaining 12 patients in whom apical aneurysms were identified only by CMR, in 11 patients, the aneurysm size was small or medium, and in only 1 patient, it was large [4]. Apical aneurysms were recognized in each of the 28 patients diagnosed with CMR, and all the aneurysms identified by echocardiography were confirmed by CMR [4]. The low sensitivity of echocardiography has been confirmed by another study in apical HCM, where the rate of missed diagnosis was more than 50%, mostly due to small aneurysms (<20 mm) [32]. Thus, echocardiography seems to have high specificity but low sensitivity in detecting LV aneurysms, mostly those of small or medium size. To increase the sensitivity of echocardiography, sonographers should always keep in mind the foreshortening of the LV apex. Foreshortening occurs when the ultrasound beam does not cut through the true LV apex but transects above and anterior to the true apex. This geometric distortion of the LV cavity and walls makes the apex look “rounded” instead of the normal “bullet” shape. As a result, the long axis of the left ventricle appears shorter, and the false apex is thicker and apparently hyper contractile. Consequently, there will be an overestimation of both the global and regional LV function and an underestimation of the LV volume and length (Figure 7). In addition, apical thrombus, infarction or aneurysm can be missed in this view. Foreshortening can be corrected by moving the probe more toward the apex and laterally (Figure 7).

Patients with MVO and apical aneurysms may not have high mid LV gradients both at rest and during the Valsalva maneuver; in fact, MVO, caused by complete systolic apposition of the LV walls, frequently occurs without high velocities. This is due to the complete cessation or diminution of the flow caused by the complete emptying and by akinesia of the apex, further decreasing the flow through the severely narrowed mid ventricular cavity [8]. Consequently, in the absence of flow, there are no high velocities, even in the presence of visually severe obstruction at 2D echocardiography. This phenomenon represents a potential source of confusion, leading to an incorrect categorization of patients as non-obstructed, even though complete emptying can be seen in echocardiographic views. In a series of 40 symptomatic HCM patients with suspected obstruction who underwent cardiac catheterization, Malcolmson et al. [33] demonstrated that in 40% of patients, there was severe obstruction at catheterization and no Doppler gradient at echocardiography. They attributed the false-negative rate and lower gradients to the cessation of the Doppler flow in the mid–left ventricle. In addition, they concluded that recognition of abrupt mid-systolic flow cessation in symptomatic HCM patients should suggest an invasive assessment of LV gradients. The observation of low velocities has been confirmed in HCM patients with MVO and LV aneurysm. Sherrid et al. found that 95% of HCM patients with LV aneurysm had mid LV obstruction with mid LV complete systolic emptying. Of the patients with obstruction, 84% had a mid-systolic Doppler signal void, a marker of complete flow cessation, but only 19% had Doppler systolic gradients ≥ 30 mm Hg [8]. In the absence of aneurysm, patients with apical–mid HCM and complete emptying at the apex showed Doppler patterns of complete emptying or LV high velocities without a drop.

The observation that, in the presence of MVO, Doppler echocardiography could underestimate gradients, further underestimated by the presence of LV aneurysm, raises the question of whether an invasive assessment is indicated, at least in symptomatic patients with such anatomic and functional abnormalities. The AHA guidelines state that echocardiography remains the gold standard for non-invasive assessment of obstruction, so there is no rationale to perform invasive hemodynamic evaluation, at least in the routine assessment of patients with obstructive HCM [9]. However, catheterization invasive studies are recommended in selected cases where diagnostic information is not obtained by a non-invasive test and could influence management.

**Figure 8:**
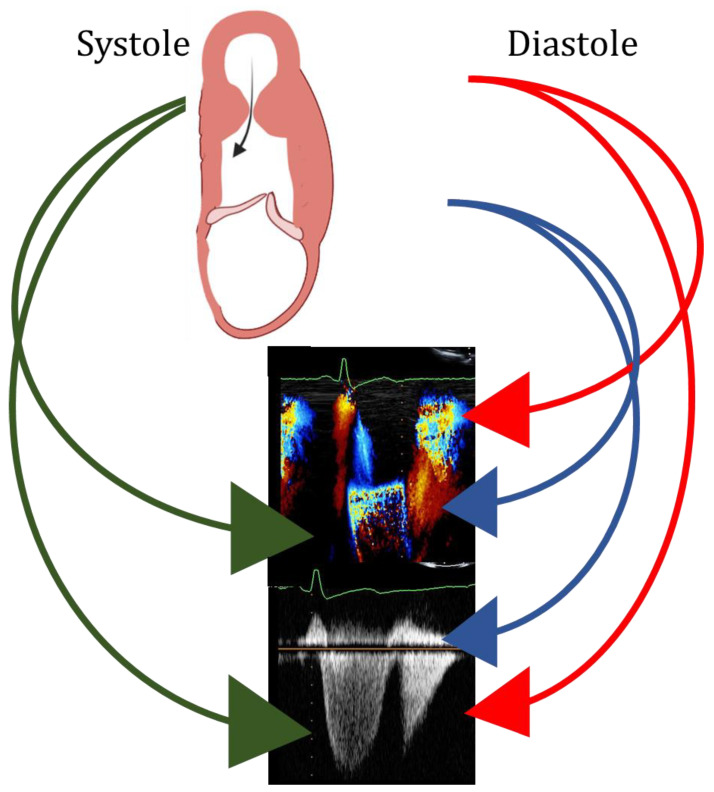
Upper panel: scheme of left ventricular mid ventricular obstruction and apical aneurysm in systole (left) and diastole (right). Green arrows: systolic flow at color (mid panel) and at continuous Doppler (bottom panel), indicating left ventricular obstruction. Blue arrows: early peak diastolic antegrade flow at color (mid panel) and at continuous Doppler (bottom panel), indicating left ventricular filling. Red arrows: early peak diastolic retrograde flow at color (mid panel) and at continuous Doppler (bottom panel), indicating diastolic gradient between the apex and the mid portion of the left ventricle.

The published data suggest that, during the execution of echocardiography in HCM, sonographers and/or clinicians should search for the presence of a LV aneurysm, particularly in patients with apical and/or mid LV hypertrophy. As reported above, the first correct approach is to avoid foreshortening when attempting to capture the true apex. Thereafter, correct orientation, i.e., parallel to flow, of the Doppler cursor is mandatory to detect not only the highest velocities by continuous Doppler, but also, by pulsed Doppler, the LV region where gradients develop. The anteriorization and medialization of the continuous Doppler line serve to exclude mitral regurgitation from the LV outflow tract. In addition, M-Mode color Doppler imaging offers a unique opportunity to study intra-ventricular flows during the cardiac cycle in patients with LV aneurysm in whom even diastolic gradients could be identified [34] (Figure 8).

The use of contrast echocardiography has been suggested to increase sensitivity in detecting not only LV aneurysm but also thrombus. The AHA and ESC guidelines give a Class 2a recommendation to use contrast echocardiography in case of suboptimal images or in case of apical HCM and/or when an aneurysm is suspected [9,12]. However, as reported above, CMR is superior to echocardiography in detecting the presence of aneurysms and showing transmural LGE, consistent with myocardial fibrosis and scarring at the level of LV aneurysmatic wall [2,4,5].

## 4. Management

LV aneurysm in HCM has been linked to an increased risk of adverse cardiovascular outcome [4,7,35,36,37,38,39,40]. A recent meta-analysis has shown that, among six studies comprising 2382 HCM patients, LV apical aneurysm was associated with an increased risk of thromboembolic and SCD events [7]. According to this meta-analysis, LV aneurysm should be included in the stratification algorithm for SCD [7], and patients with HCM and LV aneurysm should be evaluated for anticoagulation. Although the study is of great interest, it presents some limitations. A considerable proportion of the patient population comes from, and the challenging definition of adverse outcome because of both low prevalence of LV aneurysm in the HCM population and the relatively short follow-up periods. Below, we will discuss the evidence/suggestions for HCM patients with LV aneurysm.

Anticoagulation. To date, the pathogenesis of LV thrombus is based on Virchow’s triad of thrombogenesis: stasis attributable to reduced ventricular function, endocardial injury and inflammation/hypercoagulability. The relative contribution of each of these factors to LV thrombus formation depends on the cause of the myocardial dysfunction and its duration. Although regional endocardial injury and inflammation may be the dominant factors after an acute MI, stasis attributable to regionally reduced LV function may be the key factor in HCM with LV aneurysm. In fact, in HCM, the presence of an apical aneurysm is associated with an increased risk of apical thrombus/thromboembolic events due to dyskinesia/akinesia of the endocardium [2,3,4,5,6,7,8]. However, despite this risk, there is no consensus on prophylactic anticoagulation in patients with aneurysm without evidence of thrombus. Looking at data derived from MI, which are certainly wider than those derived from HCM, the presence of an aneurysm or an extensive anterior MI is not clearly indicative of prophylactic anticoagulation [38]. No published randomized trials specifically address full-dose anticoagulation for the prevention of LV thrombus among patients with MI in the percutaneous coronary intervention era. In addition, studies of antiplatelet and anticoagulant combination therapy in patients with other indications for oral anticoagulant have demonstrated a several-fold increased risk of bleeding [38]. Data coming from dilated non-ischemic cardiomyopathy (DCM) demonstrated that thrombus is less frequently diagnosed compared with ischemic cardiomyopathy. Consequently, there are few data on the incidence of LV thrombus and subsequent thromboembolic events in patients with DCM [38]. In a few studies performed in HCM, the thromboembolic risk increases linearly with aneurysm size, and a cut-off >2 cm has been identified as particularly predictive of thromboembolic events [2,3,4,5,6,7,8,18,19,20,21,22,23,24].

**Figure 9:**
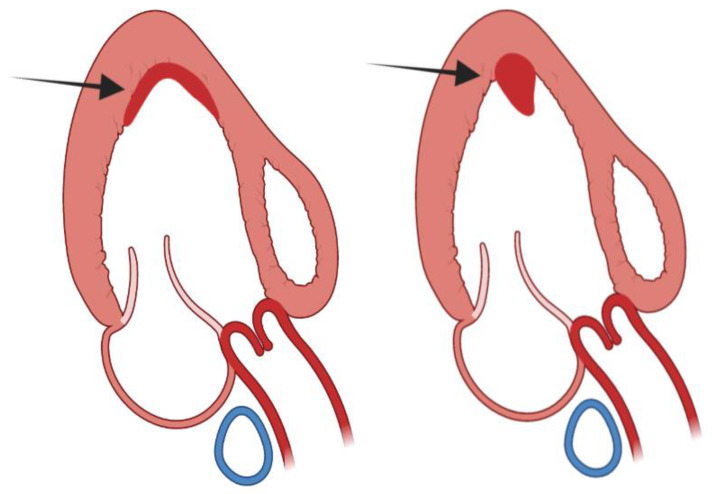
**Left** panel: mural or sessile left ventricular thrombus; **right** panel: protuberant or sessile left ventricular thrombus.

After acute MI, the formation of LV thrombus is associated with a 5.5-fold increased risk of embolic events compared with no thrombus. The magnitude of benefit with good anticoagulation control (commonly defined when warfarin is used with time in therapeutic range ≥70%) likely outweighs the potential increased risk of bleeding among patients with LV thrombus, even in the presence of antiplatelet therapy. Even in these cases, limited evidence suggests that anticoagulation therapy is more likely to resolve LV thrombus and to lower embolic risk compared with no or subtherapeutic anticoagulation [38]. In the presence of thrombus, imaging also helps in the morphological classification of thrombus itself—mural or sessile—in case the borders are contiguous with the adjacent endocardium (Figure 9), protuberant or pedunculated in case the borders are distinct from the adjacent endocardium and it protrudes into the LV cavity (Figure 9). Mural thrombi are more difficult to diagnose with echocardiography, and if suspected, contrast echocardiography or CMR are indicated. The risk of embolization has always been linked to sessile thrombus, although embolization has been found in some series in up to 40% of patients with mural thrombosis [39]. Thus, the recent statement from AHA suggests that it would be prudent to anticoagulate newly diagnosed mural thrombus [38]. If mural thrombus persists after a course of oral anticoagulation therapy, particularly if it is organized or calcified, the risk of embolization is likely low, and in such cases, a shared decision-making approach seems appropriate, weighing the small but likely non-zero risks of cardioembolic stroke against the risks of oral anticoagulation therapy [38]. Concerning the treatment of LV thrombus, the same statement affirms that direct oral anticoagulant therapy is an alternative to vitamin K antagonist, particularly in patients in whom the therapeutic range of INF is difficult to achieve. The duration of treatment is another aspect not well addressed by current data; however, there is a consensus about a re-evaluation after 1–3 months from the first diagnosis. Although thrombus persistence despite ongoing anticoagulation and recurrence after completion of anticoagulation are understudied conditions, prolonged anticoagulation and repeated imaging assessment are generally recommended [38].

**Figure 10:**
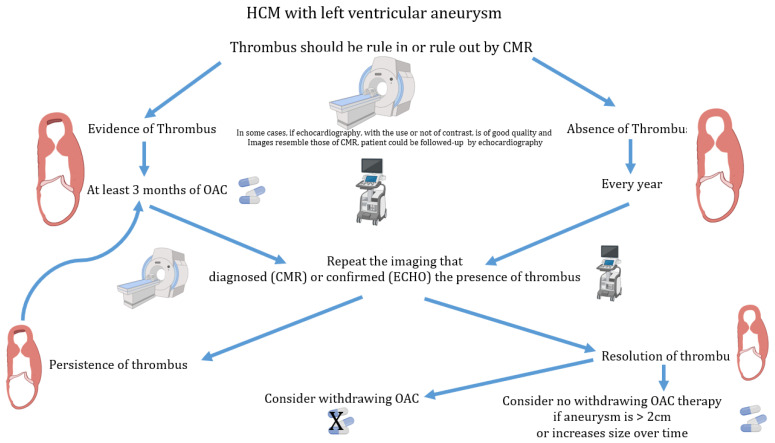
Flow chart for the management of anticoagulation therapy in patients with hypertrophic cardiomyopathy and left ventricular aneurysm. CMR = cardiac magnetic resonance; Echo = echocardiography; HCM = hypertrophic cardiomyopathy; OAC = oral anticoagulation.

Considering these data, we propose a flow chart for the management of patients with HCM and LV aneurysm with and without evidence of thrombus (Figure 10).

Major arrhythmias. The presence of a LV aneurysm appears to be a negative prognostic factor with a higher risk of occurrence of potentially fatal ventricular tachyarrhythmia events compared to HCM patients without aneurysm [41]. This is further corroborated by the fact that appropriate ICD interventions for monomorphic ventricular tachycardia (VT)/ventricular fibrillation (VF) are more frequent in patients in whom an ICD has been implanted for primary prevention based on the presence of a LV apical aneurysm, thus recommending its implantation for primary prevention [2,3,4,5,6,7,8]. This evidence corroborates the idea that aneurysm represents an area of myocardium with extensive fibrosis, which constitutes an anatomical substrate for the generation of malignant tachyarrhythmias (mainly though re-entry mechanisms) and increased SCD risk [42,43,44,45,46]. Myocardial ablation with radiofrequency at the edge of the aneurysm in some case eliminates sustained ventricular tachyarrhythmias [47]. In HCM patients without aneurysm, advancing age is associated with a lower risk of SCD, whereas in subjects with aneurysm, advancing age is not associated with a reduction in risk [12]. It is presumable that aneurysms and the contiguous fibrotic scar, arrhythmogenic focus, develop or progress in the patient’s natural history, unlike other HCM markers for which the risk seems to decrease with age.

The role of LV aneurysm in the stratification of risk of sudden death is different between the European and USA guidelines. The American College of Cardiology and the American Heart Association guidelines have established that apical aneurysm is a risk modifier for SCD in HCM, concluding that prophylactic ICD implantation is a reasonable choice in these patients [9], whereas the ESC guidelines suggested that there are insufficient data to support the introduction of apical aneurysms as a criterion for guiding ICD therapy in HCM [12] (Table 1). However, in the recent 2022 ESC Guidelines for the management of patients with ventricular arrhythmias and the prevention of sudden cardiac death, the presence of LV aneurysm is considered in class IIa for indication of ICD. Thus, the ESC guidelines are still unclear about the role of LV aneurysm in SCD risk stratification [48] (Table 1)**.**

Surgical therapy. Few data exist on the outcome after resection of LV aneurysm. One report from the group of the Mayo Clinic, Rochester, suggests that surgical resection of LV aneurysms should be considered in severely symptomatic patients with HCM. The authors reported that among 44 treated patients, the early mortality risk was acceptable, and the operation appeared to reduce the overall risk of adverse events, especially ventricular arrhythmias and embolic stroke [49].

**Table 1:** Indication of ICD implantation in patients with HCM and LV ventricular aneurysm. ACC = American College of Cardiology; AHA = American Heart Association; ESC = European Society of Cardiology; HRS = Heart Rhythm Society; ICD = Implantable cardiac defibrillator; LV = Left ventricular.

Guidelines	Indication for ICD Implantation in Presence of LV Aneurysm	Class of Recommendation and Level of Evidence
2014 ESC Guidelines on diagnosis and management of Hypertrophic Cardiomyopathy [12]	No specific indication in presence of LV aneurysm.	
2017 AHA/ACC/HRS Guideline for Management of Patients with Ventricular Arrhythmias and the Prevention of Sudden Cardiac Death [50]	LV aneurysm may be associated with a risk of sustained monomorphic VT, and ICD implantation is appropriate.	Class II Level A
2020 AHA/ACC Guideline for the Diagnosis and Treatment of Patients with Hypertrophic Cardiomyopathy [9]	LV aneurysm independent of the size is included in the major risk factors.	Class 1 Consensus of expert opinion based on clinical experience
2022 ESC Guidelines for the management of patients with ventricular arrhythmias and the prevention of Sudden Cardiac Death [48]	LV aneurysm is included in the major risk factors.	Class II Level B

## 5. Conclusions

LV aneurysm in HCM is not a frequent finding. CMR has higher sensitivity than echocardiography in the diagnosis of LV aneurysm, confirming that this imaging technique should be performed in all patients with HCM. The mechanisms of LV aneurysm formation are linked to the complex pathophysiology of the disease. There is no universal agreement on the role of LV aneurysm in the stratification of sudden death risk, while there is more agreement on the use of anticoagulation therapy when large LV aneurysms are detected.

## Data Availability

Not applicable.
